# Ocular squamous cell carcinoma in cattle: clinicopathological and prognostic considerations

**DOI:** 10.29374/2527-2179.bjvm011025

**Published:** 2026-06-15

**Authors:** Ananda Teixeira Neves Pontes, Maria Katarina Lopes Cabral, Camylla Monteiro Meneses, Luis Teles Coutinho, José Augusto Bastos Afonso da Silva, Maria Isabel de Souza, Márcia Bersane Araújo de Medeiros Torres

**Affiliations:** 1 Programa de Pós-graduação em Saúde Animal, Universidade Federal do Agreste de Pernambuco (UFAPE), Garanhuns, PE, Brazil; 2 Universidade Federal do Agreste de Pernambuco, Garanhuns, PE, Brazil; 3 Clínica de Bovinos de Garanhuns, Universidade Federal Rural de Pernambuco, Garanhuns, PE, Brazil

**Keywords:** ruminant diseases, skin diseases, eye diseases, keratinocytes, neoplasms, doenças de ruminantes, doenças de pele, doenças oculares, queratinócitos, neoplasias

## Abstract

Ocular and periocular neoplasms in cattle are of great clinical and economic significance, with squamous cell carcinoma (SCC) being the most prevalent. The aim of this study was to analyze the occurrence of neoplasms in these regions in cattle from the Agreste region of Pernambuco by evaluating 45 cases (25 retrospective and 20 prospective), including clinical, epidemiological, and morphological data. SCC was the most frequent tumor (n = 43), with one case of papilloma also observed. In addition, one ocular dermoid, a non-neoplastic lesion, was identified. SCC was mainly located in the third eyelid, with lesions smaller than 3 cm, exclusively affecting adult females of dairy breeds. Microscopically, according to a multifactorial classification system, grade 1 tumors were predominant. The presence of multinucleated and basal cells was noted in neoplasms with relatively aggressive biological behavior. In the prospective cases, clinical progression could be followed, with records of recurrence and economic losses for farmers. Neoplasms larger than 3 cm were associated with relatively poor biological behavior, suggesting the importance of early clinical diagnosis for prognosis. The results reinforce the relevance of incorporating epidemiological, clinical, and morphological factors into lesion staging, aiming to improve the grading system used in this study for application to ocular carcinomas in cattle.

## Resumo

As neoplasias oculares e perioculares em bovinos são de grande importância clínica e econômica, destacando-se o carcinoma de células escamosas (CCE). Objetivou-se com esse estudo analisar a ocorrência de neoplasias destas regiões em bovinos do Agreste de Pernambuco, por meio da análise de 45 casos (25 retrospectivos e 20 prospectivos), incluindo seus dados clínicos, epidemiológicos e morfológicos. O CCE foi o tumor mais frequente (n=43), com um caso de papiloma também observado. Adicionalmente, foi identificado um dermoide ocular, uma lesão não neoplásica. O CCE estava localizado principalmente na terceira pálpebra, com lesões menores que 3 cm, acometendo exclusivamente fêmeas adultas de raças leiteiras. Microscopicamente, predominaram tumores grau 1, conforme classificação multifatorial. Foi observada a presença de células multinucleadas e células basais em neoplasias de pior comportamento biológico. Nos casos prospectivos, foi possível acompanhar a evolução clínica, com registro de recidivas e perdas econômicas dos produtores. As neoplasias maiores que 3 cm foram associadas ao pior comportamento biológico, sugerindo a importância do diagnóstico clínico precoce para o prognóstico. Os resultados reforçam a importância da adição de fatores epidemiológicos, clínicos e morfológicos para estadiamento das lesões visando aperfeiçoar o sistema de graduação usado neste estudo para a aplicação em carcinomas oculares em bovinos.

## Introduction

Cattle farming plays a central role in the Brazilian economy. In addition to being one of the country’s main agricultural activities, it supports extensive production chains, thereby generating employment and income across different regions (Malafaia et al., 2021). However, several diseases can compromise animal productivity and welfare, such as neoplasms affecting the ocular and periocular regions. These conditions cause significant economic and sanitary losses as they impair vision, reduce reproductive capacity, lead to progressive weight loss, increase treatment costs, and may result in carcass condemnation (Carvalho et al., 2012; Schulz & Anderson, 2010).

Squamous cell carcinoma (SCC) is the most common malignant epithelial neoplasm affecting the bovine ocular region. Its occurrence is multifactorial, involving environmental factors such as solar radiation exposure, genetic predisposition related to breed and lack of pigmentation on the eyelids, and viral infections by bovine papilomavírus (Fornazari et al., 2017; Heeney & Valli, 1985; Karakurt et al., 2023; Quevedo et al., 2020; Tsujita & Plummer, 2010).

Clinicopathological evaluation of this neoplasm is essential to determining prognosis and guide therapeutic decisions. In veterinary medicine, the most widely used histological classification is that described by Weiss and Frese (1974), primarily based on the degree of cellular differentiation and keratinization. The histological classification proposed by Nagamine et al. (2017) introduces more comprehensive multifactorial criteria, incorporating assessment of the invasion pattern, mitotic index, and the intensity of the histiolymphocytic infiltrate (Corrêa et al., 2021).

Therefore, studies that integrate clinical, epidemiological, and morphological data are crucial to elucidating disease progression. Therefore, the present study aimed to characterize ocular and periocular neoplasms in cattle, with emphasis on squamous cell carcinoma, correlating clinicopathological findings and discussing potential prognostic implications.

## Materials and methods

The study was conducted using 45 samples with suspected ocular and periocular neoplasms from the Agreste mesoregion of the State of Pernambuco, Brazil. The samples were obtained from animals treated at the Clínica de Bovinos de Garanhuns (CBG) da Universidade Federal Rural de Pernambuco (UFRPE), between 2009 and 2025, and from the routine diagnostics of the Setor de Patologia Veterinária (SPV) do Laboratório de Anatomia e Patologia Animal (LAPA) da Universidade Federal do Agreste de Pernambuco (UFAPE) between 2014 and 2025.

### Clinical and epidemiological data

Information recorded on the animals’ clinical forms was compiled and organized chronologically in Microsoft Excel spreadsheets. The clinical and epidemiological variables considered included age, breed, coat color, management system, city of origin, macroscopic tumor description, size, anatomical location, laterality, and associated ocular changes, when available in the clinical records. Tumor size was categorized as follows: T1 (<3 cm), T2 (3–5 cm), and T3 (>5 cm), used only as descriptive tumor size categories and not as a formal staging system. When tumor size was not recorded on the clinical form, measurements were performed at the SPV/LAPA/UFAPE considering the largest axis of the lesion, when sample integrity allowed for adequate measurement.

Animal owners included in the prospective subset and treated between August 2023 and May 2025 (n = 10) were contacted six months after lesion excision to obtain information regarding the animal’s outcome and the possible occurrence of tumor recurrence.

When information was not available in the clinical records, the variable was excluded in the corresponding analysis.

### Histological processing

Samples obtained from surgical biopsies were fixed in 10% formalin, processed using the paraffin-embedding technique, and stained with hematoxylin and eosin.

### Microscopic analysis

Cases of SCC were classified according to the multifactorial grading system proposed by Nagamine et al*.* (2017) (Table 1), which categorizes lesions as grade I, II, or III, based on the progression of malignancy criteria determined by the cumulative score of histological parameters. The remaining cases were described descriptively. Slides were examined independently by two observers at different times.

**Table 1:** Multifactorial grading system proposed by Nagamine et al. (2017).

**Morphological Feature**	Score*			
	1	2	3	4
**Degree of keratinization**	Highly keratinized (>50% of cells)	Moderately keratinized (20%–50% of cells)	Minimal keratinization (5%–20% of cells)	No keratinization (0%–5% of cells)
**Pattern of invasion**	Pushing, well-delineated infiltrating borders	Infiltrating, solid cords, bands, and/or strands	Small groups or cords of infiltrating cells (*n*> 15)	Widespread cellular dissociation in small groups and/or in single cells (*n*< 15)
**Host response**	Marked	Moderate	Slight	None
**Nuclear pleomorphism**	Little nuclear pleomorphism (>75% mature cells)	Moderately abundant nuclear pleomorphism (50%–75% mature cells)	Abundant nuclear pleomorphism (25%–50% mature cells)	Extreme nuclear pleomorphism (0%–25% mature cells)
**Mitoses per high power field**	0–1	2–3	4–5	>5

Note: The tumor grade is based on the total score as follows: Grade 1 (6–10), Grade 2 (11–15), Grade 3 (16–20). (Nagamine et al., 2017).

### Statistical analysis

Data were descriptively analyzed using the R and RStudio software. The chi-square test (χ^2^) was applied with a 95% significance level (*p* < 0.05) to evaluate correlations between the histological grade of SCC and observed variables. To identify categories contributing significantly to differences between observed and expected values, adjusted residuals were calculated; those with an absolute value ≥ 1.96 were considered significant. Chi-square test results were confirmed using Fisher’s exact test.

## Results

Of the 45 samples clinically diagnosed as ocular or periocular neoplasms in cattle from the Agreste mesoregion of Pernambuco State, 44 (97.77%) were confirmed as neoplasms, of which 43 were classified as SCC and one as papilloma. One sample corresponded to an ocular dermoid malformation. Given the marked predominance of SCC among the cases, the subsequent results and discussion focus on this neoplasm.

**Figure 1:**
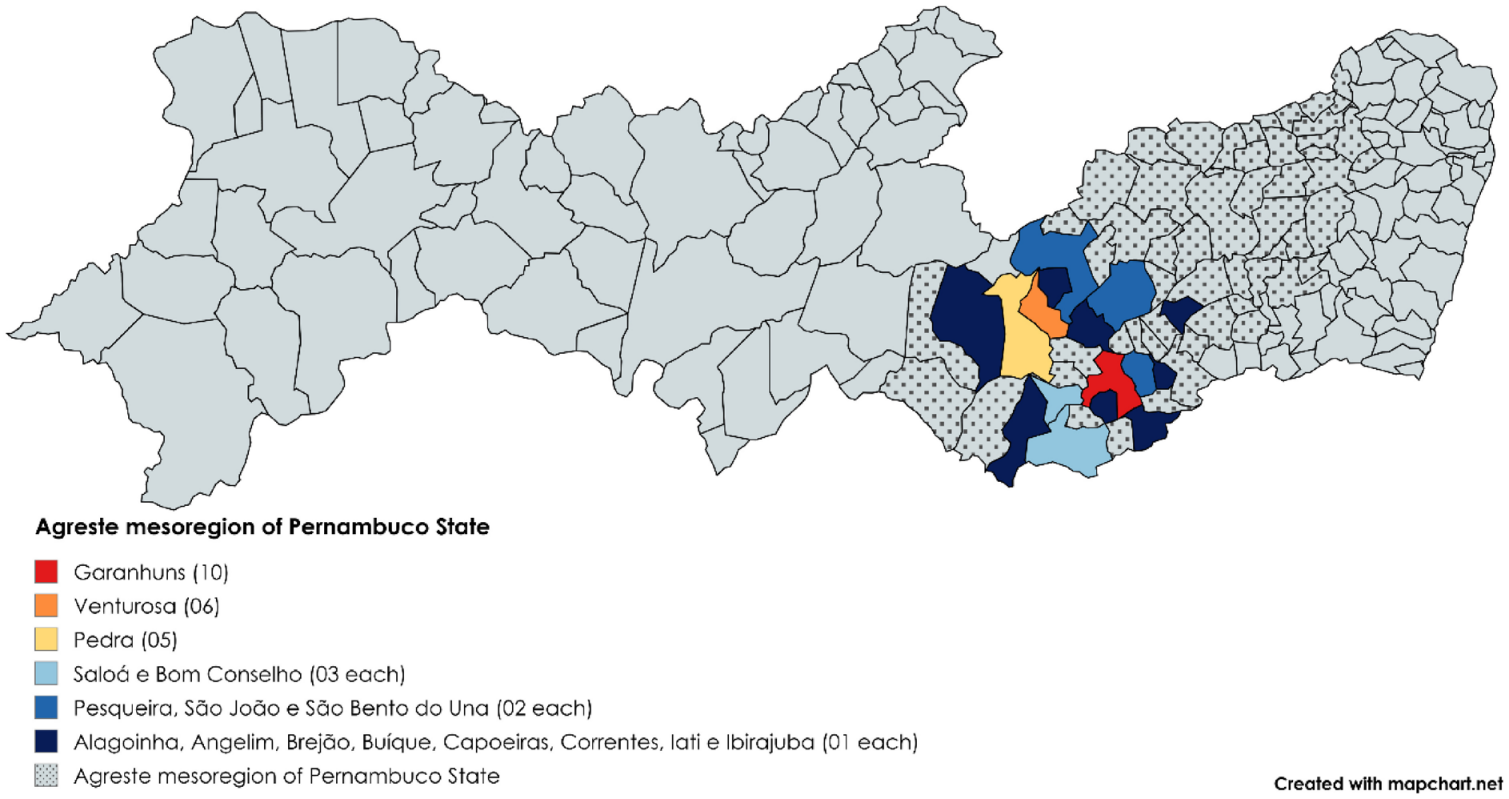
Geographic distribution of ocular and periocular squamous cell carcinoma samples in cattle from the Agreste mesoregion of the State of Pernambuco, Brazil.

The geographic distribution of the 43 SCC cases showed the highest frequency in the municipality of Garanhuns (23.25%), with the remaining cases distributed among other municipalities within the mesoregion (Figure 1). In two cases, the origin of the sample was not reported.

All 43 affected animals were females, aged between 2 and 10 years (mean = 5.5 years). Regarding the management system, the semi-intensive regime predominated (n = 23; 53.48%), followed by intensive (n = 9; 20.93%) and extensive (n = 3; 6.97%) systems. In eight cases (18.18%), this information was not recorded. Purebred Holstein cattle (n = 20; 46.51%) and Holstein crossbreeds (n = 19; 44.18%) were predominant, followed by Girolando (n = 3; 6.97%) and one animal of undefined breed (2.32%). The black-and-white coat pattern was the most frequently observed (n = 38; 88.37%).

Lesions were mainly located in the periocular region (n = 34; 79.06%), thus predominantly affecting the third eyelid (n = 27; 62.79%), followed by combined involvement of the third and lower eyelids (n = 5; 11.62%), the upper eyelid (n = 1; 2.32%), and the lower eyelid (n = 1; 2.32%). Nine cases (20.93%) involved the ocular region. The right eye was more frequently affected (n = 24; 55.81%), followed by the left eye (n = 15; 34.88%), with three bilateral cases (6.97%) and one with no information available (2.32%).

Regarding tumor size, 16 cases (37.20%) were classified as T1, 11 as T2 (25.58%), and four as T3 (9.30%); in 12 cases (27.90%), this information was not available.

The most common treatment was tumor excision (n = 20; 46.51%), followed by enucleation (n = 15; 34.88%), third eyelid removal (n = 4; 9.30%), and a combination of tumor excision with third eyelid removal (n = 2; 4.65%). One animal was euthanized (2.32%), and in one case (2.32%), the procedure was not specified.

**Figure 2:**
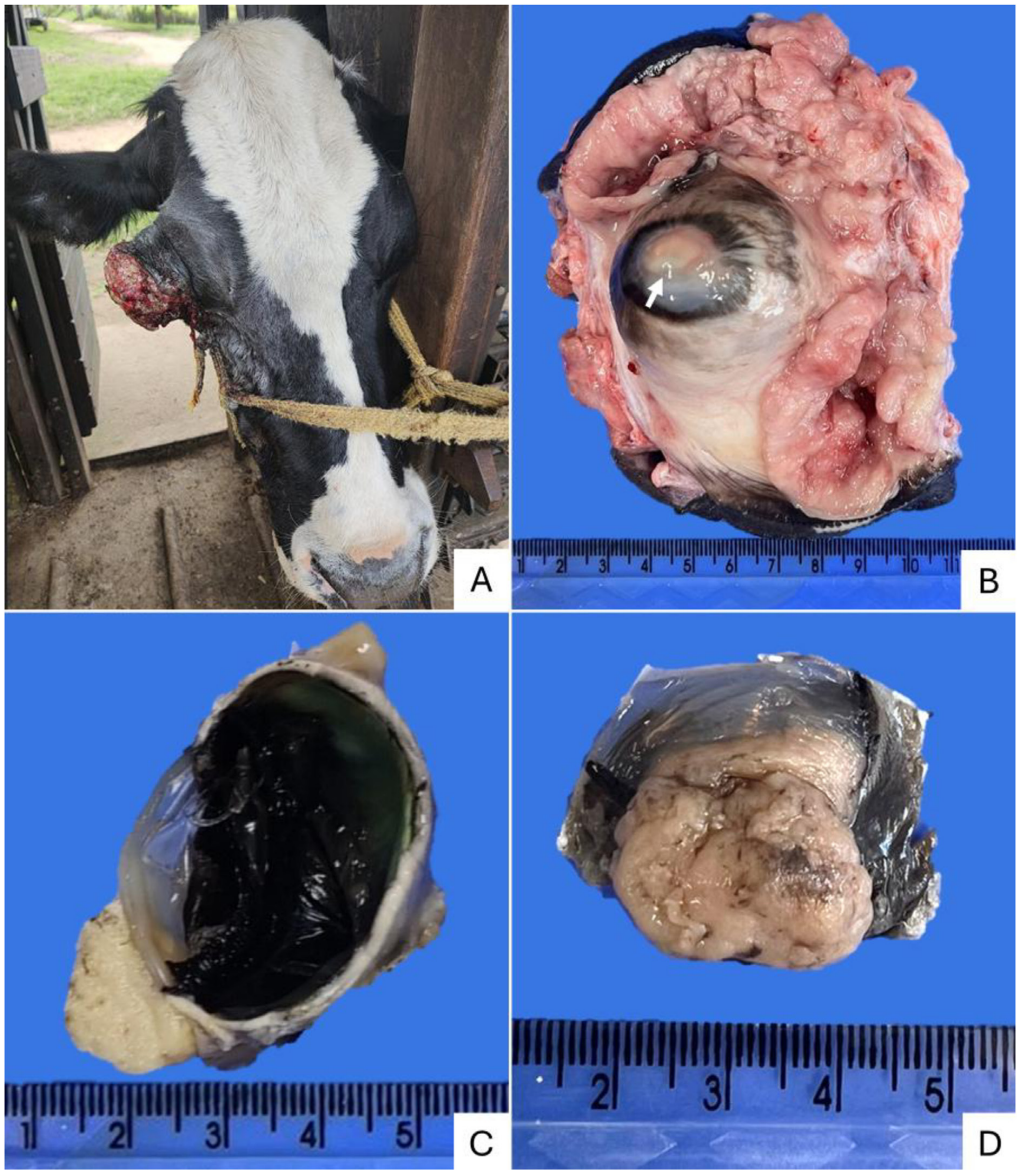
Macroscopic aspects of ocular and periocular neoplasms in cattle.

Macroscopically, the lesions appeared as irregular, papillary, exophytic, or multinodular masses of variable consistency, sometimes ulcerated and accompanied by purulent discharge, fibrin deposition, and myiasis (Figure 2).

(A) Exophytic squamous cell carcinoma (SCC) occupying the right eyeball of a bovine subjected to euthanasia. (B) SCC affecting both lower and upper eyelids, measuring 17 cm in circumference, and associated with a corneal ulcer (arrow). (C) SCC located on the eyeball with an arboriform appearance, adherent to the cornea, measuring 3 × 1.5 cm. (D) SCC with an irregular surface invading the cornea, measuring 2.5 × 1.5 cm in diameter. **Source:** Clínica de Bovinos de Garanhuns (CBG/UFRPE) (2025).

Of the SCC cases evaluated, 42 could be graded according to the system proposed by Nagamine et al. (2017); only one case was diagnosed as SCC in situ, for which grading was not applicable. Among the graded cases, 20 (47.61%) were classified as grade I, 18 (42.85%) as grade II, and four (9.52%) as grade III. The parameters assessed in this system included the degree of keratinization, invasion pattern, inflammatory response, nuclear pleomorphism, and mitotic count per field (Table 2).

**Table 2:** Location, size, and histological grading according to Nagamine et al. (2017) for bovine squamous cell carcinoma (n = 42).

Grade	Location		Size			
	Ocular	Periocular	T1	T2	T3	NI*
Grade I	06	14	12	4	0	4
Grade II	03	15	4	4	4	6
Grade III	0	4	0	2	0	2

* NI: Not informed.

T1: < 3 cm. T2: 3–5 cm. T3: > 5 cm.

**Figure 3:**
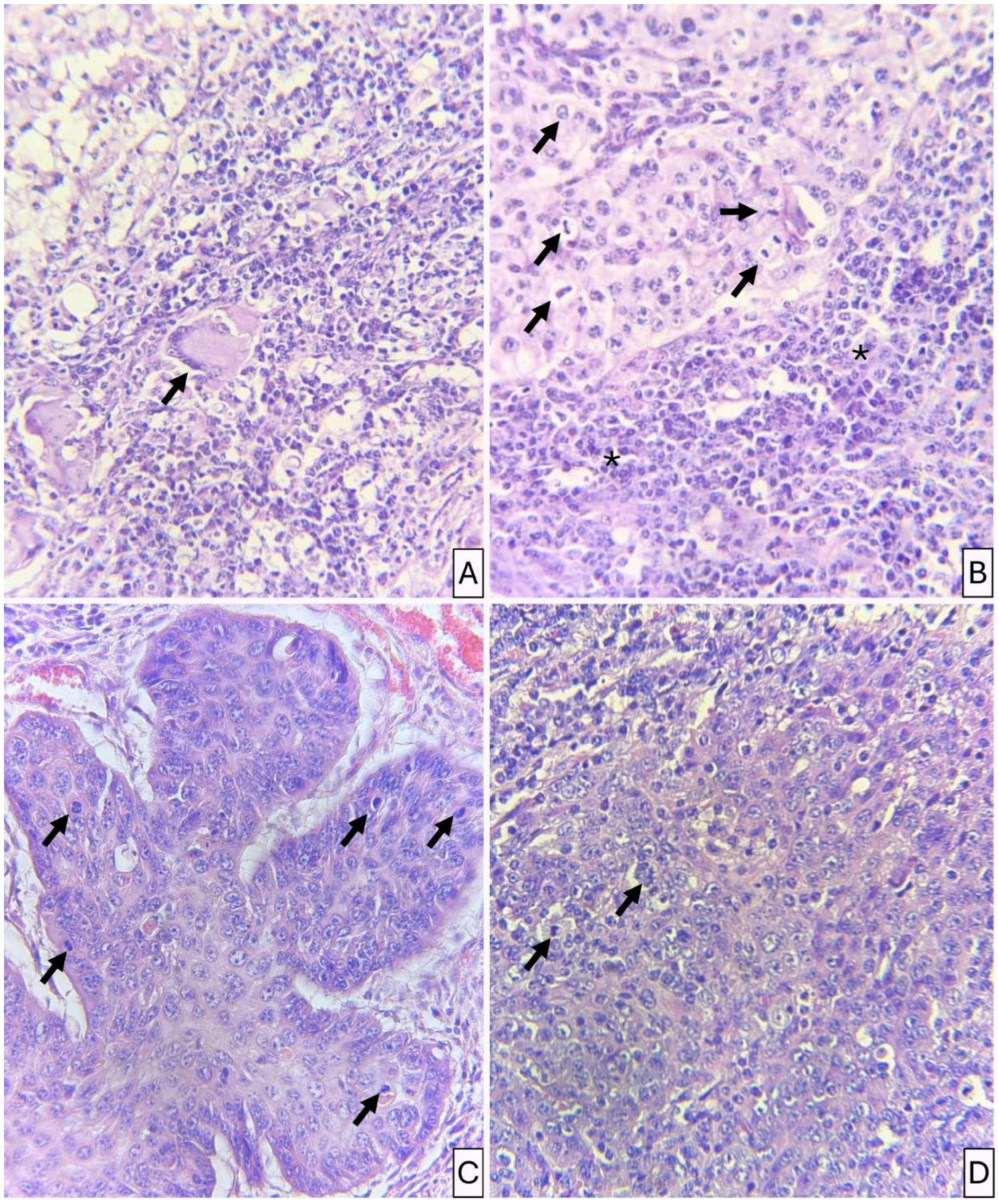
Histological photomicrographs of bovine ocular squamous cell carcinomas.

Among the evaluated SCC cases, four exhibited specific histological variations in their morphology: two cases of papillary squamous carcinoma (Figure 3C) and two showing an acinar pattern.

(A) Grade III squamous cell carcinoma (SCC) showing a multinucleated cell. Hematoxylin and eosin (H&E), 400× magnification. (B) Grade III SCC showing basal cells (asterisks) and mitotic figures in keratinocytes (arrows). H&E, 400× magnification. (C) Papillary carcinoma, grade II, showing atypical mitotic figures (arrow). H&E, 400× magnification. (D) Grade III SCC with marked nuclear pleomorphism and atypical mitotic figures. H&E, 400× magnification.

No significant association was found between histological grade and anatomical location (*p* > 0.05). However, a significant association was observed between histological grade and tumor size (*p* < 0.05), with grade I tumors occurring predominantly in smaller lesions, while higher-grade tumors (grades II and III) were more frequent in larger lesions.

Microscopically, regarding the morphological parameters proposed in the classification system, the neoplasms were predominantly organized in an infiltrative invasion pattern, forming solid cords, bands, and/or cell bundles, which corresponded to a considerable proportion of cases (72.09%; n = 31), with keratinization ranging from moderate to absent. The associated lymphoplasmacytic inflammatory infiltrate within the stromal component varied from moderate to marked. Nuclear pleomorphism ranged from moderate to marked, whereas mitotic activity was generally low, with fewer than one mitotic figure per field in a total area of 2.37 mm^2^.

In the four grade III cases, keratinization was absent, with marked pleomorphism, predominance of small infiltrative cell groups or cords, and more than five mitoses per field (Figure 3D). The inflammatory infiltrate ranged from moderate (n = 3) to marked (n = 1).

Additionally, eight cases showed the presence of multinucleated cells (Figure 3A), and eight presented basal cell proliferation (Figure 3B). Areas of necrosis and hemorrhage, as well as three cases showing invasion into the cartilage of the third eyelid, were also observed. These findings were often associated with lesion extension and local reactive changes, such as pseudoepitheliomatous hyperplasia, actinic keratosis, and hypertrophy and hyperplasia of the lacrimal glands.

A significant association was found between the presence of multinucleated cells and histological grade (*p* < 0.05). The adjusted residual analysis indicated that multinucleated cells were more frequently observed in grade III tumors (residual > 1.96). The presence of basal cells did not show a statistically significant association with histological grade (*p* > 0.05). However, the adjusted residual analysis suggested a trend toward higher occurrence in grade III tumors and lower occurrence in grade I tumors.

Among the ten cases followed after surgical treatment, six showed no recurrence, whereas four developed recurrence within less than six months. Of the recurrent cases, two animals were sent for slaughter, and two were left on the farm.

## Discussion

The results demonstrate the predominance of SCC among ocular and periocular neoplasms, thereby corroborating the findings of Martins and Barros (2014) and Vasconcelos et al*.* (2023). Similarly, in a retrospective Brazilian study, 88 ocular and periocular tumors were described in cattle, 87 of which were classified as SCC (Lucena et al., 2011). In this study, a greater number of samples originated from the municipality of Garanhuns, which, although not among those with the largest cattle herds, is located in the Agreste mesoregion, an area that concentrates a significant portion of Pernambuco’s cattle herd and milk production (IBGE, 2023a, 2023b). This result is likely related to the location of the study site, where the samples were received.

The mean age observed (5.5 years) aligns with that reported by Kayapinar et al. (2023) and Quevedo et al. (2020). The predominance of cases in adult cattle may be associated with prolonged exposure to environmental carcinogenic agents such as ultraviolet radiation, a factor linked to the etiopathogenesis of cutaneous neoplasms (Pfeifer, 2020). Physiological changes associated with aging, including alterations in the immune system and cellular repair mechanisms, may also contribute to a greater vulnerability to tumor development (Tsujita & Plummer, 2010). These aspects reinforce the influence of age on the carcinogenesis process.

The exclusive involvement of females, also noted by Gharagozlou et al. (2007), is associated with their longer lifespan in dairy herds compared with males used for beef production. As with age, sex influences the duration of exposure to environmental risk factors owing to the animals’ zootechnical purpose. Therefore, sex, age, and production purpose constitute interrelated factors that contribute to the higher occurrence of ocular and periocular SCC (Tsujita & Plummer, 2010).

Although authors such as Sousa et al. (2011) reported a higher occurrence of SCC in extensively managed cattle, the predominance of the semi-intensive system observed here reflects the main management model in the study region, thereby corroborating the description by Souza et al. (2023). According to these authors, the origin area of the animals corresponds to the state’s main dairy basin, characterized by a predominance of reproductive-age and lactating females.

Similarly to Quevedo et al. (2020), Fornazari et al. (2017), and Lucena et al. (2011), who reported a predominance of Holstein cattle, the wide presence of this breed in regional dairy farming may explain its representation among the observed cases. Other studies in different regions have identified greater involvement of breeds such as Simmental, Nellore, and Hereford (Kayapinar et al., 2023; Sousa et al., 2011; Tsujita & Plummer, 2010). Moreover, skin depigmentation is described as a predisposing factor contributing to greater ultraviolet exposure (Gharagozlou et al., 2007; Tsujita & Plummer, 2010).

The higher occurrence of lesions in the third eyelid, also reported by Fornazari et al. (2017) and Quevedo et al. (2020), is inconsistent with that reported by other authors, who described this structure as less frequently affected, with the most common sites being the conjunctiva, palpebral conjunctiva, and corneoscleral junction (Gharagozlou et al., 2007; Kayapinar et al., 2023; Martins & Barros, 2014). The findings of this study highlight the anatomical relevance of the third eyelid as a preferential site for periocular SCC. The higher frequency of neoplasms in the right eye followed a pattern similar to that reported in other studies (Fornazari et al., 2017; Gharagozlou et al., 2007; Kayapinar et al., 2023). Despite this recurrence, conclusive scientific explanations for this apparent predisposition are lacking.

Regarding tumor size, well-established criteria for using tumor dimensions as a prognostic indicator in cattle are lacking; this is in contrast with tumor dimensions in other species, where larger tumor size is associated with more malignant behavior and a higher occurrence of metastases (Gedon et al., 2021; Santos et al., 2023). Brazilian sanitary legislation, under Article 165 of Decree No. 10,468/2020 (Brasil, 2020), mandates the condemnation of carcasses with extensive neoplasms, regardless of metastasis or general condition. Therefore, further studies are needed to establish a relationship between tumor size and malignancy, thus allowing the development of a size-based classification applicable to cattle and reducing subjective evaluations and economic losses.

Surgical excision is the most recommended treatment, particularly for tumors smaller than 3 cm (Tsujita & Plummer, 2010). Although not the most frequent procedure, enucleation, performed in 34.88% of cases, is relevant because it directly affects animal welfare owing to its invasive nature and entails higher costs for producers.

The macroscopic features of SCC lesions were similar to those reported in other studies (Carvalho et al., 2012; Fornazari et al., 2017; Gharagozlou et al., 2007). The lack of association between histological grade and tumor location may have been influenced by the anatomical proximity of ocular and periocular structures. In contrast with studies on other species, no studies on cattle have reported a relationship between anatomical site and prognosis.

Regarding histological variations, the differentiation between papillary squamous carcinoma and papilloma followed the histopathological criteria described by Bao et al. (2012), emphasizing malignant features such as invasion and atypical mitoses.

The presence of moderate to marked inflammation in grade III neoplasms observed in this study contrasts with the findings of Nagamine et al. (2017), who reported that greater inflammatory intensity is associated with better prognosis. Similarly, in dogs, inflammation did not influence tumor differentiation (Santana et al., 2016). These findings reinforce the paradoxical nature of inflammation within the tumor microenvironment, which may either represent an antitumor response or be modulated by neoplastic cells toward a pro-tumoral profile, thus contributing to tumor progression and suppression of the host immune response (Figueiredo, 2019).

Multinucleated cells derived from the neoplastic lineage may indicate more aggressive biological behavior and poorer prognosis (Khan, 2022). Conversely, when derived from macrophages, they may represent an antitumoral response and better clinical outcomes (Gessain et al., 2024). Although determining the origin of these cells in this study was impossible, their association with relatively high histological grade may represent an additional marker of malignancy.

The tendency toward a higher occurrence of basal cells in higher-grade tumors suggests that basal cell proliferation may be related to relatively aggressive neoplasms, consistent with literature linking this finding to the progression of preneoplastic lesions into invasive SCC (Falkenberg et al., 2023).

Follow-up of the animals allowed the identification of recurrences and the observation of possible associations between tumor grade and both macroscopic and microscopic features, suggesting the potential relevance of tumor size and histological grade as prognostic indicators. It also enabled the assessment of related impacts, including animal welfare and economic consequences for producers. From a production perspective, promoting animal welfare adds value to animal-derived products, meeting current consumer and international market demands for production systems ensuring welfare throughout the animal’s life cycle up to slaughter (Malafaia et al., 2021).

## Conclusions

SCC in cattle represents the main ocular and periocular neoplasm in this species, and clinical-epidemiological factors influence the occurrence of the disease. The multifactorial grading system proposed by Nagamine et al. (2017) contributed to a more optimized morphological evaluation and correlation with the analyzed scores for the assessment of SCC in this location. Additionally, findings such as the presence of multinucleated cells and basal cell proliferation may be considered potential prognostic markers for this neoplasm. Further studies may support the development of a specific staging system for SCC in this location and species, with potential implications for clinical management and sanitary regulations.
